# Dietary coconut water vinegar for improvement of obesity-associated inflammation in high-fat-diet-treated mice

**DOI:** 10.1080/16546628.2017.1368322

**Published:** 2017-09-21

**Authors:** Nurul Elyani Mohamad, Swee Keong Yeap, Huynh Ky, Wan Yong Ho, Sook Yee Boo, Joelle Chua, Boon-Kee Beh, Shaiful Adzni Sharifuddin, Kamariah Long, Noorjahan Banu Alitheen

**Affiliations:** ^a^ Department of Cell and Molecular Biology, Faculty of Biotechnology and Biomolecular Science, Malaysia Agricultural Research and Development Institute (MARDI), Serdang, Malaysia; ^b^ China-ASEAN College of Marine Sciences, Xiamen University Malaysia, Sepang, Malaysia; ^c^ Department of Agriculture Genetics and Breeding, College of Agriculture and Applied Biology, Can Tho University, Can Tho City, Vietnam; ^d^ School of Biomedical Sciences, University of Nottingham Malaysia Campus, Semenyih, Malaysia; ^e^ Science Vision Sdn Bhd, Shah Alam, Malaysia; ^f^ BioEasy Sdn Bhd, Shah Alam, Malaysia; ^g^ Institute of Bioscience, Universiti Putra Malaysia, Serdang, Malaysia; ^h^ Biotechnology Research Centre, Malaysian Agricultural Research and Development Institute (MARDI), Serdang, Malaysia

**Keywords:** Coconut water vinegar, SREBP1, adiponectin, inflammation, gut microbiota

## Abstract

Obesity has become a serious health problem worldwide. Various types of healthy food, including vinegar, have been proposed to manage obesity. However, different types of vinegar may have different bioactivities. This study was performed to evaluate the anti-obesity and anti-inflammatory effects of coconut water vinegar on high-fat-diet (HFD)-induced obese mice. Changes in the gut microbiota of the mice were also evaluated. To induce obesity, C57/BL mice were continuously fed an HFD for 33 weeks. Coconut water vinegar (0.08 and 2 ml/kg body weight) was fed to the obese mice from early in week 24 to the end of week 33. Changes in the body weight, fat-pad weight, serum lipid profile, expression of adipogenesis-related genes and adipokines in the fat pad, expression of inflammatory-related genes, and nitric oxide levels in the livers of the untreated and coconut water vinegar-treated mice were evaluated. Faecal samples from the untreated and coconut water vinegar-treated mice (2 ml/kg body weight) were subjected to 16S metagenomic analysis to compare their gut microbiota. The oral intake of coconut water vinegar significantly (*p* < 0.05) reduced the body weight, fat-pad weight, and serum lipid profile of the HFD-induced obese mice in a dose-dependent manner. We also observed up-regulation of adiponectin and down-regulation of sterol regulatory element-binding protein-1, retinol-binding protein-4, and resistin expression. The coconut water vinegar also reduced HFD-induced inflammation by down-regulating nuclear factor-κB and inducible nitric oxide synthase expression, which consequently reduced the nitric oxide level in the liver. Alterations in the gut microbiota due to an increase in the populations of the *Bacteroides* and *Akkermansia* genera by the coconut water vinegar may have helped to overcome the obesity and inflammation caused by the HFD. These results provide valuable insights into coconut water vinegar as a potential food ingredient with anti-obesity and anti-inflammatory effects.

## Introduction

Obesity is an epidemic disease caused by multiple endogenous and environmental factors. Among these factors, excessive caloric intake, particularly from energy-dense meals, is thought to be the main factor contributing to global obesity [1]. Furthermore, obesity is known as a mediator that triggers inflammation [2]. Obesity-induced inflammation always leads to other chronic diseases, including diabetes, cardiovascular disease, hypertension, and even cancer [3]. Dietary modifications, regular exercise, and a healthy lifestyle are among the common strategies for managing obesity. In terms of obesity adjuvants, orlistat and sibutramine are the only anti-obesity drugs approved by the US Food and Drug Administration (FDA). However, these drugs are associated with side effects [4]. Thus, continuous efforts are being made to identify potential ingredients that are safe for long-term consumption to manage body weight and to control serum triglycerides and total cholesterol levels in the body.

Functional food ingredients have been proposed as better choices than anti-obesity drugs, as are generally considered to be safe and do not have side effects. Nonetheless, some of the functional food ingredients that claim to have anti-obesity effects have not been scientifically validated. Soybean, for example, is a food ingredient that has been reported to have antioxidant and anti-obesity effects. In particular, fermented soybean was found to have an enhanced anti-obesity effect compared to unfermented soybean, especially in reducing body weight and adipocytes in high-fat-diet (HFD)-induced obese mice [5].

Coconut water is the aqueous part of the coconut endosperm, containing sugar, inorganic ions, vitamins, amino acids, and phytohormones. Young coconut water has been widely consumed worldwide as a refreshing beverage and for its health benefits, which are mainly contributed by its above-mentioned composition [6]. In the early days of the coconut industry, mature coconut water was normally treated as a waste by-product [7]. As coconut water contains a substantial amount of sugar [6], it is a suitable source of carbohydrate for the production of fermented beverages and vinegar [7]. Although coconut water is a nutritious beverage and has been proven to be a suitable starting material for vinegar production [6,7], the bioactivities of coconut water vinegar, particularly its anti-obesity effect, have yet to be scientifically validated. Previous studies have reported that coconut water *in vivo* possesses a hepatoprotective effect against carbon tetrachloride (CCl_4_)-induced liver damage [8–11]. In this study, coconut water was able to ameliorate CCl_4_-induced inflammation, fatty liver, and necrosis [8,12]. It has been shown that this effect is generated through the ability of the coconut water to restore the antioxidant levels in animals with CCl_4_-induced liver damage [12]. In addition, the antioxidant effect of coconut water restored insulin sensitivity and the anti-hypertension effect in fructose-fed hypertensive rats [13]. A 2016 report showed that coconut water was able to reduce adipose tissue mass by activating a metabolic action through the attenuation of leptin levels in animals fed high-fat and high-fructose diets [14]. As coconut water contains a high amount of sugar [6], it is suitable as a starting material for fermentation. Vinegar is a food ingredient produced via the alcoholic and acetic fermentations of its carbohydrate origins. Traditionally, it has been used as a seasoning, a preservative, and an ingredient for condiments [15]. Vinegar has been reported to possess various bioactivities, including anti-bacterial, anti-obesity, anti-insulin resistance, and anti-tumour effects [16]. However, the bioactivities and health benefits of vinegar may vary owing to the different origins of the carbohydrate and the microbes used during the fermentation process [17]. Coconut water was reported to activate lipid metabolism and to have an anti-inflammatory effect [14]. However, to our knowledge, the anti-obesity effect of coconut water vinegar has never been tested. Thus, this study aimed to evaluate the anti-obesity, antioxidant, and gut microbiota population alteration effects of coconut water vinegar when fed to HFD-induced obese mice.

## Materials and methods

### Coconut water vinegar

Coconut water was purchased from Pasar Borong Selangor (Seri Kembangan, Malaysia). It first underwent anaerobic fermentation with *Saccharomyces cerevisiae 7013 INRA* (MARDI Culture Collection Centre, Serdang, Malaysia) at room temperature for 7–10 days to produce alcohol. The alcohol was then further fermented with *Acetobacter acetii vat Europeans* (MARDI Culture Collection Centre, Malaysia) at room temperature under aerobic conditions for 4 weeks to produce coconut water vinegar that standardises to 7.0 ± 1.0% acetic acid detected by reverse-phase chromatography [18]. Then, the coconut water vinegar was stored in a stainless-steel container for 30 days for the maturation process. Finally, the matured coconut water vinegar was filtered using a 1 mm pore size stainless-steel strainer, diluted to 4% acidity and stored in a glass bottle at 4°C until used. Gallic acid (97.05 ± 2.43 µg/ml), vanillic acid (107.78 ± 4.67 µg/ml), and 4-hydroxybenzoic acid (77.48 ± 2.11 µg/ml) were detected in the coconut water vinegar [18].

### Animals

This work was approved by the Institutional Animal Care and Use Committee (IACUC), Universiti Putra Malaysia (UPM) (UPM/IACUC/AUP-R045/2016), and was performed in accordance with the Guide for the Care and Use of Laboratory Animals prepared by the IACUC, UPM. Twenty-four male C57BL/6 mice (7 weeks old) were purchased from Monash University Malaysia Campus, Malaysia. The mice were acclimatised for 7 days in plastic cages at room temperature (22 ± 1°C) with 12 h dark/light cycles, and fed a standard pellet diet and distilled water *ad libitum*.

### Experimental design

The mice were divided into three groups, with eight mice in each group (Table 1). An HFD of D12451 (45 kcal% fat; Research Diets, New Brunswick, NJ, USA) was used to induce obesity in all the mice for 33 weeks continuously. Low-dose [CL, 0.08 ml/kg body weight (BW)] and high-dose (CH, 2 ml/kg BW) groups were given coconut water vinegar daily via oral gavage, starting from week 24 until week 33. Untreated obese control mice (UT) were given distilled water only. The CH dose was selected based on the previous *in vivo* study of vinegar [19,20], while the CL dose was selected based on one tablespoon of vinegar in 250 ml of water, which is the common daily vinegar intake [19]. Body-weight measurements were taken once every week. At the end of the treatment, the mice were killed by intraperitoneal injection of ketamine and xylazine (120 mg/kg) (Sigma, St Louis, MO, USA), followed by cervical dislocation. Then, blood was collected by cardiac puncture using a 23 g needle and 1 ml syringe. The liver and white adipose tissues (gonadal fat pad) were then collected and placed in a 10 ml centrifuge tube containing 2 ml of phosphate-buffered saline (Sigma-Aldrich, USA) before further analysis.

**Table 1.** Grouping of the high-fat-diet (HFD) induced obese mice according to treatment.

Group	Treatment
UT (untreated obese control)	Induced obese mice given HFD + distilled water (control)
CL (low-dose coconut water vinegar)	Induced obese mice given HFD + 0.08 ml/kg body weight coconut water vinegar
CH (high-dose coconut water vinegar)	Induced obese mice given HFD + 2 ml/kg body weight coconut water vinegar

### Biochemical profile

The collected blood was separated using BD Microtainers® (BD, San Diego, CA, USA). The obtained sera were then diluted 10× and tested for cholesterol, triglycerides (TG), and high-density lipoprotein (HDL) using a biochemical analyser (Hitachi 902 Automatic Analyzer, Roche, Grenzach-Wyhlen, Germany) and adapted reagents from Roche (Grenzach-Wyhlen, Germany). Low-density lipoprotein (LDL) was calculated using the Friedewald equation: LDL-cholesterol (mmol/l) = Cholesterol − HDL − (TG)/2.2.

### Gene expression analysis using quantitative polymerase chain reaction (qPCR)

The messenger RNA (mRNA) expression of the adipose and liver tissues was determined using real-time quantitative polymerase chain reaction (qPCR), according to the methods described in a previous study [19]. In brief, the mRNA from the gonadal fat pad or liver was extracted using an RNeasy Mini Kit (Qiagen, Hilden, Germany). One microgram of the total RNA was then reverse transcribed to the first strand complementary DNA (cDNA) using the iScript™ Reverse Transcription Supermix forqPCR (Bio-Rad, Hercules, CA, USA), according to the manufacturer’s protocols. qPCR was performed with the iTaq™ Universal SYBR® Green Supermix (Bio-Rad, USA). The quantities of the target genes and the housekeeping genes were calculated according to a standard curve, and their expression was measured using iQ5 Software (Bio-Rad, USA). The expression levels in all the sample groups were compared with the untreated control group, and the levels of different mRNAs in the untreated control group were designated as 1. All of the results were expressed as fold changes and were measured in triplicate. Non-template controls were used to confirm the specificity. The primer sequences used in the study are given in Table 2.

**Table 2.** Primer sequences used in the quantitative real-time polymerase chain reaction assay.

**Target genes**	
AD	Forward: 5ʹ-TCAGGAAGAGGAGGAGGA-3ʹ
	Reverse: 5ʹ-TCAGGAAGCACATCATACG-3ʹ
SREBP1c	Forward: 5ʹ-TCATCAACAACCAAGACAGT-3ʹ
	Reverse: 5ʹ-CCAGAGAAGCAGAAGAGAAG-3ʹ
GLUT4	Forward: 5ʹ-CTGCTTCTGGCTCTCACA-3ʹ
	Reverse: 5ʹ-AGGACATTGGACGCTCTC-3ʹ
iNOS	Forward: 5ʹ-GCACCGAGATTGGAGTTC-3ʹ
	Reverse: 5ʹ-GAGCACAGCCACATTGAT-3ʹ
NF-κB	Forward: 5ʹ-CATTCTGACCTTGCCTATCT-3ʹ
	Reverse: 5ʹ-CTGCTGTTCTGTCCATTCT-3ʹ
**Reference genes**	
GAPDH	Forward: 5ʹ-TTCCAGCCTTCCTTCTTG-3ʹ
	Reverse: 5ʹ-GGAGCCAGAGCAGTAATC-3ʹ
ACTB	Forward: 5ʹ-GAAGGTGGTGAAGCAGGCATC-3ʹ
	Reverse: 5ʹ-GAAGGTGGAAGAGTGGGAGTT-3ʹ
HPRT	Forward: 5ʹ-CGTGATTAGCGATGATGAAC-3ʹ
	Reverse: 5ʹ-AATGTAATCCAGCAGGTCAG-3ʹ

AD, adiponectin; SREBP1c, sterol regulatory element-binding protein-1c; GLUT4, glucose transporter type 4; iNOS, inducible nitric oxide synthase; NF-κB, nuclear factor-κB; GAPDH, glyceraldehyde-3-phosphate dehydrogenase; ACTB, beta-actin; HPRT, hypoxanthine phosphoribosyltransferase.

### Determination of nitric oxide (NO) level in liver homogenate

The assay was performed following the manufacturer’s protocols for the Griess reagent assay (Invitrogen, Waltham, MA, USA). In brief, 150 μl of the liver homogenate was mixed with 20 μl of Griess reagent and 130 μl of distilled water in a 96-well plate. The mixture was then incubated for 30 min and measured at 540 nm using an enzyme-linked immunosorbent assay Plate Reader (Bio-Tek Instruments, Winooski, VT, USA).

### Proteome profiler assay (adipokine)

The effect of coconut water vinegar on the secreted cytokines from the adipose tissue was analysed using an adipokine proteome profiler kit (R&D Systems, Minneapolis, USA). In brief, the membranes were blocked using a blocking buffer at room temperature for 1 h. Concurrently, the protein samples were incubated with the detection antibody at room temperature for 1 h. Next, the protein samples coupled with the detection antibody were added to the membrane and incubated at 4°C overnight. The next day, the membranes were washed three times using a washing buffer and incubated with streptavidin–horseradish peroxidase for 30 min. Finally, the membranes were washed a further three times before the substrate was added to develop a chemiluminescence condition. The membrane was then viewed using a ChemiDoc XRS (Bio-Rad, USA).

### Metagenomic study

A high-concentration coconut water vinegar faecal sample was selected to undergo further metagenomic analysis. Faecal samples from week 33 (end treatment) were collected and kept at −80°C until further use. On the selected day, the faecal samples were mashed in liquid nitrogen using a pestle and baked overnight. The faecal samples were weighed, and the DNA sample was extracted using a QIAamp DNA stool mini kit (Qiagen, Hilden, Germany), according to the manufacturer’s protocol. The DNA sample was purified using Agencourt® AMPure® XP beads (Beckman Coulter, Brea, CA, USA), and the concentration was measured using a Qubitfluorometer (Thermo Fisher Scientific, Waltham, MA, USA). The 16S amplicon in the V3 and V4 regions was amplified in the first PCR (forward primer: 5′-CGTCGGCAGCGTCAGATGTGTATAAGAGACAGCCTACGGGNGGCWGCAG-3′; reverse primer: 5′-GTCTCGTGGGCTCGGAGATGTGTATAAGAGACAGGACTACHVGGGTATCTAATCC-3′) [21]. In brief, 25 µl of the DNA sample at a concentration of 12.5 ng of DNA was mixed with 12.5 μl of 2× KAPA Hifi Hot Start Ready Mix (Kapa Biosystems, Wilmington, MA, USA), and 1 μM of 16S amplicon PCR forward and reverse primers. The PCR was set to 95°C for 3 min, followed by 35 cycles of 30 s at 95°C, 30 s at 55°C, and 30 s at 72°C. The quality check for the first PCR product was carried out by purifying the product using Agencourt AMPure XP beads (Beckman Coulter, USA) then running them through with the agarose gel to confirm the correct size before the second PCR commenced. The second PCR amplification was conducted by adding the product from the first PCR with the unique barcode using the Nextera XT index kit (Illumina, San Diego, CA, USA). In the second PCR amplification, 5 µl of the first PCR product, 25 μl of 2× KAPA Hifi Hot Start Ready Mix, and Nextera XT index Primer 1 and 2 were mixed together. The PCR was set to 95°C for 3 min, followed by eight cycles of 30 s at 95°C, 30 s at 55°C, and 30 s at 72°C, and finally, 5 min at 72°C. The quality check for the second PCR product was carried out using Agencourt AMPure XP beads and an Agilent Bioanalyzer 2100 (Agilent Technologies, Santa Clara, CA, USA). Based on the results of the Bioanalyzer, the concentrations of the products were normalised to 4 nM and pooled together. The pooled samples were then prepared using the standard library protocols to a final concentration of 9 pM and run on the MiSeq Sequencer (2 × 300 bp paired end reads) (Illumina) using a 600-cycle MiSeq V3 reagent (Illumina). The 16S metagenomic analysis was performed using MEGAN (MEtaGenome-ANalyzer) based on a comparison of the BLASTX against the National Center for Biotechnology Information (NCBI) database.

### Statistical analysis

All of the quantitative measurements are presented as the mean ± SD. The analysis was performed using one-way analysis of variance (ANOVA), and the group means were compared by Duncan’s test. A *p* value < 0.05 was considered statistically significant.

## Results

### Body weight and fat-pad weight changes

**Figure 1.**
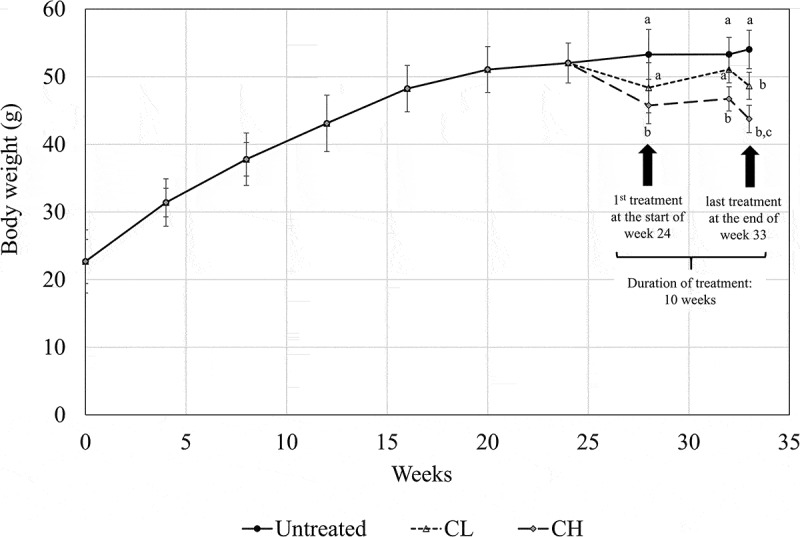
Body weight measurement (week 0–33) of untreated mice and mice fed 0.08 ml/kg body weight (BW) coconut water vinegar (CL) or 2 ml/kg BW coconut water vinegar (CH). Data are shown as the average of biological replicates of mice from the same treatment group. Different letters indicate significant differences among groups (*p* < 0.05).

In this study, a drastic average weight gain, from approximately 22 g to 52 g, was recorded in the mice after being fed for 24 weeks on an HFD. A subsequent 10 weeks of HFD further increased the body weight of the control mice only to approximately 53 g (Figure 1). Ten weeks of coconut water vinegar treatment led to approximate reductions of 17.9% and 8.7% in body weight for CL (0.08 ml/kg BW) and CH (2 ml/kg BW), respectively, compared to the untreated mice (Table 3).

**Table 3.** Body weight, fat-pad weight, and fat-pad/body weight ratio of untreated (UT), 0.08 ml/kg body weight (BW) coconut water vinegar (CL) and 2 ml/kg BW coconut water vinegar (CH)-treated high-fat-diet (HFD)-induced obese mice at the end of week 33.

Group	Final BW (g/mouse)	Fat-pad weight	Fat-pad/BW ratio (%)
UT	53.30 ± 0.50^a^	2.43 ± 0.14^a^	4.56 ± 0.07^a^
CL	48.64 ± 1.74^a^	2.11 ± 0.04^a^	4.82 ± 0.05^a^
CH	43.76 ± 1.46 ^b^	1.31 ± 0.25^b^	2.54 ± 0.49^b^

Data are shown as mean ± SD of biologically replicated mice from the same treatment group.

Different superscript letters indicate significant differences among the groups (*p* < 0.05).

### Serum lipid profile assay

In association with the reduction in body weight, the HFD-induced obese mice treated with coconut water vinegar showed a reduction in the levels of serum cholesterol, triglycerides, and LDL. In contrast, the HDL level and the ratio of HDL/LDL in the coconut water vinegar-treated groups increased compared to the untreated group (Table 4). This hypolipidaemic effect of the coconut water vinegar was recorded in a dose-dependent manner.

**Table 4.** Serum biochemical profile of untreated (UT), 0.08 ml/kg body weight (BW) coconut water vinegar (CL) and 2 ml/kg BW coconut water vinegar (CH)-treated high-fat-diet (HFD)-induced obese mice.

Group	Cholesterol (mmol/l)	Triglyceride (mmol/l)	LDL (mmol/l)	HDL (mmol/l)	HDL/LDL
UT	5.21 ± 0.15^a^	6.27 ± 0.71^a^	1.26 ± 0.12^a^	1.10 ± 0.15^a^	0.87 ± 0.13^a^
CL	3.71 ± 0.46^b^	2.95 ± 0.55^b^	0.95 ± 0.11^a^	1.72 ± 0.12^a^	1.81 ± 0.09^a^
CH	3.67 ± 0.21^b^	2.39 ± 0.22^c^	0.75 ± 0.02^b^	3.03 ± 0.11^b^	3.20 ± 0.15^b^

Data are shown as mean ± SD of biologically replicated mice from the same treatment group.

LDL, low-density lipoprotein; HDL, high-density lipoprotein.

Different superscript letters indicate significant differences among the groups (*p* < 0.05).

### Expression of Glut4, adiponectin, and SREBP genes in the adipose tissue

**Figure 2.**
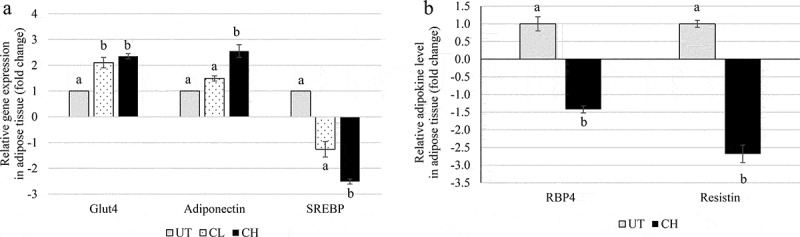
(a) Quantitative polymerase chain reaction analysis of obesity-related genes [glucose transporter type 4 (Glut4), adiponectin, and sterol regulatory element-binding protein-1 (SREBP1)] of untreated (UT) mice and mice fed 0.08 ml/kg body weight (BW) coconut water vinegar (CL) or 2 ml/kg BW coconut water vinegar (CH). (b) Significant adipokines down-regulated by coconut water vinegar treatment in high-fat-diet obese mice tested using adipokine proteome profiler analysis. RBP4, retinol-binding protein-4. Data are presented as the mean ± SD of biological replicates of mice from the same treatment group. Different letters indicate significant differences among groups (*p* < 0.05).

In the adipose tissues of the coconut water vinegar-treated groups, the mRNA expression of glucose transporter type 4 (GLUT4) and adiponectin was up-regulated in a dose-dependent manner, compared to the untreated group. On the other hand, the expression of sterol regulatory element-binding protein (SREBP1) was down-regulated in the coconut water vinegar-treated groups, compared to the untreated group (Figure 2).

### Level of adipokines in adipose tissue

Among all the adipokines, retinol-binding protein-4 (RBP4) and resistin were the two major adipokines that were significantly down-regulated in the adipose tissues of the obese mice treated with 2 ml/kg BW of coconut water vinegar, compared to the untreated obese mice (Figure 2(b)).

### NO level and NF-κB and iNOS expression in the liver

**Figure 3.**
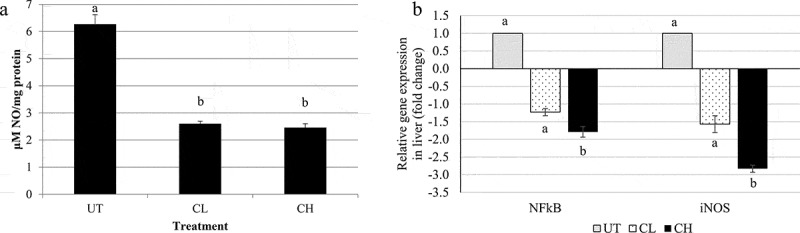
(a) Nitric oxide (NO) content and (b) quantitative polymerase chain reaction analyses of inflammation related genes [nuclear factor-κB (NFκB) and inducible nitric oxide synthase (iNOS)] of untreated (UT) mice and mice fed 0.08 ml/kg body weight (BW) coconut water vinegar (CL) or 2 ml/kg BW coconut water vinegar (CH). Data are presented as the mean ± SD of biologically replicated mice from the same treatment group. Different letters indicate significant differences among groups (*p* < 0.05).

Both coconut water vinegar-treated groups showed similar reductions in the level of NO in the liver when compared to the untreated group (Figure 3(a)). This effect was correlated with down-regulated the expression of nuclear factor-κB (NF-κB) and inducible nitric oxide synthase (iNOS) genes in the liver of the coconut water vinegar-treated groups, compared to the untreated group (Figure 3(b)).

### Alteration of gut microbiota

**Figure 4.**
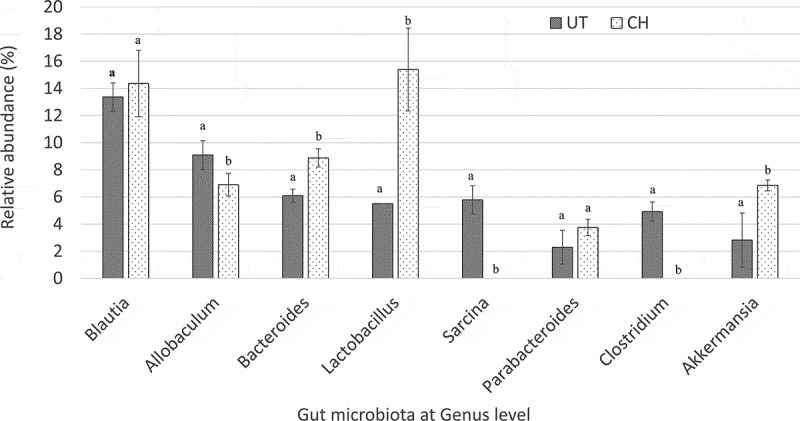
Comparison of the relative abundance of gut microbiota at genus level of untreated (UT) mice and mice fed 2 ml/kg body weight coconut water vinegar (CH) at the end of week 33 post-experiment using Illumina 16S rRNA metagenomic sequencing. Data are presented as the mean ± SD of biologically replicated mice (*n* = 6) from the same treatment group. Different letters indicate significant differences between groups (*p* < 0.05).

In the 16S metagenomic sequencing analysis of faecal samples, *Blautia* was the most dominant genus detected among the gut microbes in both the untreated and 2 ml/kg BW coconut water vinegar-treated groups. The populations of *Lactobacillus*, *Bacteroides*, and *Akkermansia* genera in the faecal samples of the coconut water vinegar-treated group were 3, 1.4, and 2.4 times higher, respectively, than in the untreated group. On the other hand, pathogenic genera, including *Sarcina* and *Clostridium* genera, were present only in the untreated group but not in the coconut water vinegar-treated groups. The population of the *Allobaculum* genus was slightly lower in the coconut water vinegar-treated groups than in the untreated group (Figure 4).

## Discussion

Consumption of an HFD, which is normally associated with a high energy density, has been identified as one of the major causes of obesity [22]. Previous studies have reported that HFD-induced obese animals are pathologically similar to obese humans and, thus, are suitable models for anti-obesity studies [23,24]. Similarly to the finding by Williams et al. [23], C57Bl/6 mice fed with an HFD were observed to have a drastic increase in body weight until week 20, reaching an average body weight of 50 g per mouse. After that, it was observed that the body weight of the untreated HFD-induced obese mice increased slightly until week 33. Besides body weight, the untreated obese mice were also shown to have a high percentage of fat-pad weight per body weight and a high serum lipid profile. Treatment with both 0.08 ml/kg and 2 ml/kg BW (CL and CH, respectively) of coconut water vinegar reduced the body weight and improved the serum lipid profile of the HFD-induced obese mice in a dose-dependent manner. A previous study, by Seo et al. [16], reported on the anti-obesity and hypolipidaemic effects of tomato vinegar. Based on their findings, tomato vinegar (14 ml/kg BW) was able to reduce the body weight by 14.3% and the serum triglycerides by 19.1% after 6 weeks of treatment. However, the tomato vinegar did not alter the total serum cholesterol in the obese mice. In this study, treatment with 2 ml/kg BW (CH) of coconut water vinegar reduced the body weight by 17.9%, the total serum cholesterol by 29.55%, and the serum triglycerides by 29.88% after 10 weeks of treatment. Previous studies have reported that coconut water possesses an anti-hypolipidaemic effect on alloxan-induced diabetes [25], cholesterol-fed [26], and fat–cholesterol-enriched-diet obese animals [27]. In addition, the duration of treatment for coconut water vinegar in this study was 4 weeks longer than in the previous report of tomato vinegar. Thus, the higher efficacy of coconut water vinegar than tomato vinegar may be accounted for by the difference in starting material [25–27] and the longer duration of treatment. The microbial strains used for the vinegar fermentation may also contribute to the observed difference in the bioactivities of these vinegars [17], but this requires further study. As coconut water vinegar ameliorated the body weight and serum lipid profile of the obese subjects, regulation of adipogenesis, inflammation, and the gut microbial population were further investigated to understand the possible regulation of coconut water vinegar in obese subjects.

Obesity is always related to excessive adipogenesis, which is regulated by the differential expression of adipogenic transcription factors, adipokines, and glucose transporters [28,29]. SREBP is a transcription factor that plays an important role in mediating transcriptional control of adipogenesis. SREBP expression results in an accumulation of lipid droplets, cholesterol homoeostasis, and an increase in high levels of the serum lipid profile [28], as observed in the untreated obese mice in this study. Coconut water vinegar, especially at a concentration of 2 ml/kg BW, significantly suppressed the expression of SREBP in the adipose tissues compared to the untreated HFD-induced obese mice. Down-regulation of SREBP gene expression may directly contribute to the reduction of the serum lipid profile, particularly the total cholesterol and triglyceride levels of the coconut water vinegar-treated obese mice. Adipokines are adipose-derived secreted factors that positively or negatively regulate adipogenesis and inflammation [30]. RBP4 and resistin are two adipokines with pro-adipogenesis effects [31,32]. A previous study reported that resistin is a suitable therapeutic target to treat high serum LDL levels [33]. In this study, the expression of both RBP4 and resistin was down-regulated in the coconut water vinegar-treated obese mice. Thus, the targeting effect of coconut water vinegar on RBP4 and resistin clearly indicated its effectiveness in ameliorating obesity and in assisting in the reduction of serum LDL levels. On the other hand, adiponectin is an adipokine with an inverse correlation with RBP4 and resistin. Adiponectin negatively regulates adipogenesis by enhancing the metabolism of energy [28,34]. Glut4 is the insulin-regulated glucose transporter found primarily in adipose tissue. Down-regulation of Glut4 in mice was reported to be associated with hepatic lipid production, which subsequently contributed to fat accumulation and insulin resistance [29]. Activation of the expression of both Glut4 and adiponectin by the coconut water vinegar treatment may help to increase the translocation and uptake of glucose in obese mice [35], increase lipid metabolism in the liver [29], and thus contribute to less lipid accumulation. Acetic acid is a major active ingredient in coconut water vinegar [18]. A previous study reported that the administration of acetic acid suppresses lipogenesis via the activation of the AMP-activated protein kinase K pathway during the metabolism of acetic acid [33]. Thus, it is likely that acetic acid plays an important role in the anti-obesity effect of coconut water vinegar.

The liver plays an important role in lipid metabolism. Its functions include supporting the lipogenesis and adipogenesis of adipocytes, thereby resulting in an excessive increase in adipose tissue [28]. Chronic obesity has been correlated with the development of liver disease owing to prolonged, mild inflammation induced by obesity [36,37]. iNOS is an inflammatory mediator that promotes the production of reactive oxygen species, NO, and endogenous signalling molecules, which contribute towards prolonging mild systemic inflammation in the liver of HFD-fed obese mice [38], which subsequently contributes to the pathogenesis of HFD-induced insulin resistance [39]. Previous studies have shown that vinegar has an anti-inflammatory effect on paracetamol-induced liver damage in mice [19]. Similarly, the treatment with coconut water vinegar was also found to reduce liver inflammation, as indicated by the down-regulation of NF-κB and iNOS gene expression and NO level in the liver. The anti-inflammatory effect of vinegar may be correlated with the presence of polyphenols and flavonoids in the vinegar [19]. A previous study reported that gallic and vanillic acids are major polyphenolic acids in vinegar [40]. Similarly to another type of vinegar, coconut water vinegar also contains gallic and vanillic acids [18]. Gallic and vanillic acids have been reported to have antioxidant, anti-inflammatory [41,42], hypolipidaemic, and liver-protective effects on HFD-induced obese rat models [42,43]. Adipokines were also correlated with obesity-related inflammation. For example, adiponectin was identified as an anti-inflammatory agent where restoration of the adiponectin hormone, which is commonly down-regulated in obese subjects, was found to inhibit obesity-induced inflammation by attenuating the activation of NF-κB [34]. Thus, other than the polyphenols, overexpression of adiponectin in the coconut water vinegar-treated mice may also have contributed to the suppression of inflammation.

It is widely accepted that the gut microbiota plays an important role in the pathophysiology of obesity [44]. An increasing population ratio of the *Blautia* genus, in the phylum Firmicutes, to the *Bacteroides* genus, in the phylum Bacteroidetes, was commonly found in the gut microbiota of obese mice. This pattern of gut microbiota further promoted inflammation via induction of the toll-like receptor-4, iNOS, and NF-κB [38]. In this study, coconut water vinegar treatment significantly increased the population of *Bacteroides* without affecting the population of the *Blautia* genus in the HFD obese mice. In addition, the populations of *Akkermansia* and *Lactobacillus* genera increased 2.5- and 3-fold, respectively, in the obese mice treated with a high dose of coconut water vinegar. The *Akkermansia* genus is negatively correlated with obesity and inflammation [44]. In terms of the *Lactobacillus* genus, different *Lactobacillus* species have been associated with positive and negative effects on the harvesting of energy and the regulation of weight [45]. Based on the draft sequencing results, *Lactobacillus johnsonii* and *L. hayakitensis* were the two most highly abundant *Lactobacillus* species found in the faecal samples collected from the coconut water vinegar-treated obese mice (results not shown). However, the genomic variations between these strains of *Lactobacillus* and their role in regulating obesity need further investigation. In contrast, the *Clostridium* genus was detected only in the faecal sample from the untreated group and not in the coconut water vinegar-treated group. Leung et al. [46] reported that obese patients, who normally have prolonged mild inflammation and slightly attenuated immunity, were more susceptible to *Clostridium* infection than the general population. Besides the *Clostridium* genus, the *Sarcina* genus, which commonly causes gastric outlet obstruction and delayed gastric emptying [47], was also only detected in the untreated group. However, the role of *Sarcina* organisms in obese subjects remains unclear and is worth further evaluation. In addition, the changes in the gut microbiota may indirectly reduce inflammation in obese mice as it controls the population of the pathogenic microbes that produce the endotoxin that induces gut inflammation [38]. Hence, the anti-inflammatory effect of coconut water vinegar may also benefit from alterations in the gut microbes.

In summary, this study showed that coconut water vinegar, which contains acetic acid, gallic acid, and vanillic acid [18], possesses anti-obesity, hypolipidaemic, and anti-inflammatory effects. This effect may have contributed to the regulation of expression of adipogenic transcription factors, adipokines, glucose transporter, and inflammatory mediators. In addition, the alteration in the gut microbiota by the coconut water vinegar treatment indirectly helped to improve lipid metabolism and reduce obesity-induced inflammation. More studies are recommended to investigate further the anti-obesity effect of coconut water vinegar by evaluating the interaction of active ingredients in the coconut water vinegar with the lipid metabolism and changes in gut microbial populations.
